# Research on food-related chronic diseases in Latin America and the Caribbean: Are we building the evidence for gender-equitable approaches?

**DOI:** 10.26633/RPSP.2019.43

**Published:** 2019-06-07

**Authors:** Teralynn Ludwick, Daniela Neri

**Affiliations:** 1 International Development Research Centre International Development Research Centre, Ottawa Ontario Canada International Development Research Centre, Ottawa, Ontario, Canada.; 2 Department of Nutrition, School of Public Health, University of São Paulo, São Paulo Department of Nutrition, School of Public Health, University of São Paulo, São Paulo São Paulo Brazil Department of Nutrition, School of Public Health, University of São Paulo, São Paulo, São Paulo, Brazil.

**Keywords:** Food, nutrition, public health, noncommunicable diseases, women’s health, equity, Latin America, Caribbean region, Alimentos, nutrición en salud pública, enfermedades no transmisibles, salud de la mujer, equidad, América Latina, región del Caribe, Alimentos, nutrição em saúde pública, doenças não transmissíveis, saúde da mulher, equidade, América Latina, região do Caribe

## Abstract

**Objectives.:**

Gender continues to be largely neglected in the global response to the noncommunicable disease epidemic. The objectives of this study were to examine current practice and barriers faced by Latin American and Caribbean (LAC) researchers in addressing gender in research on healthy food environments, and to identify future topics for gender-sensitive and gender-transformative research.

**Methods.:**

This study involved: 1) a descriptive, three-part survey to investigate to what extent LAC researchers are integrating gender considerations in research for healthier food environments and 2) a participatory workshop to coproduce ideas for future gender-sensitive and gender-transformative research.

**Results.:**

Fifty-four participants, from 19 countries, attended the workshop. Of those 54, 41 of them responded to at least one section of the three-part survey, including with 26 of the 41 responding to the section on gender. Of these 26, 17 (65.4%) had collected sex-disaggregated data and 14 (53.8%) had conducted gender analysis in recent research on food environments. Few participants had integrated gender-related findings in their recommendations and solutions. Challenges included data and methodological limitations (e.g., lack of preexisting evidence, working with secondary data), knowledge and capacity gaps, subject sensitivity, and biases. Participants identified research topics for enhancing gender equity that included food preparation norms and domestic responsibilities; differential participation of women and men in food production, distribution, and retail; and employment and school policies.

**Conclusions.:**

The findings from this study suggest that gender inequity is not being well addressed in food environment research from the LAC region. The analytical framework presented here can serve as an important starting point and resource for catalyzing future gender-transformative research. Complementary efforts are needed to overcome other challenges raised by the participating researchers, including capacity gaps, resource and data limitations, and publishing barriers.

## RESUMEN

**Objetivos.:**

La perspectiva de género se sigue descuidando en gran medida en la respuesta mundial a la epidemia de las enfermedades no transmisibles. Los objetivos de este estudio fueron examinar el procedimiento actual y los obstáculos a los que se enfrentan los investigadores de América Latina y el Caribe al abordar la perspectiva de género en investigaciones sobre entornos propicios a la alimentación saludable y determinar los temas para futuras investigaciones que estén atentas a los temas de género y sean transformadoras de las cuestiones de género.

**Métodos.:**

Este estudio incluyó: 1) una encuesta descriptiva en tres partes para conocer hasta qué punto los investigadores de America Latina y el Caribe están integrando las consideraciones de género en la investigación de entornos alimentarios más saludables y 2) un taller participativo de coprodución de ideas para futuras investigaciones sensibles al género y transformadoras de género.

**Resultados.:**

Asistieron al taller 54 participantes de 19 países. De esos 54, 41 de ellos respondieron al menos una de las secciones de la encuesta en tres partes, y 26 de los 41 respondieron a la sección sobre género. De estos 26, 17 (65,4 %) habían recopilado datos desglosados por sexo y 14 (53,8 %) habían realizado análisis de cuestiones de género en investigaciones recientes sobre los entornos de la alimentación. Pocos participantes habían incorporado resultados en materia de género en sus recomendaciones y soluciones. Algunos desafíos habían sido las limitaciones de los datos y la metodología (por ejemplo, falta de evidencia previa, trabajar con datos secundarios), falta de conocimiento y capacitación, sensibilidad de las personas y sesgos. Los participantes señalaron temas de investigación para mejorar la equidad de género, como normas de preparación de alimentos y responsabilidades domésticas; la participación diferenciada de hombres y mujeres en la producción, distribución y venta al por menor de alimentos; y el empleo y las políticas escolares.

**Conclusiones.:**

Los resultados de este estudio indican que la inequidad de género no se está abordando adecuadamente en la investigación sobre el entorno de la alimentación en América Latina y el Caribe. El marco analítico que se presenta aquí puede servir como un importante punto de partida y como recurso para catalizar investigaciones futuras transformadoras de las cuestiones de género. Se necesitan esfuerzos complementarios para superar otros desafíos que plantean los investigadores participantes, como la falta de capacitación, las limitaciones de los recursos y datos, y los obstáculos para la publicación.

## RESUMO

**Objetivos.:**

O gênero continua a ser amplamente negligenciado na resposta global à epidemia de doenças não transmissíveis. Este estudo teve por objetivo examinar a conduta e as barreiras atualmente enfrentadas pelos pesquisadores da América Latina e Caribe (ALC) ao comtemplar o gênero em pesquisas sobre ambiente alimentar saudável e identificar tópicos para futuras pesquisas sensíveis ao gênero e transformativas de gênero.

**Métodos.:**

Este estudo envolveu: 1) uma pesquisa descritiva em três partes para investigar em que medida os pesquisadores da LAC estão integrando considerações de gênero na pesquisa para ambientes alimentares mais saudáveis e 2) um workshop participativo para co-produzir ideias para pesquisas futuras sensíveis a gênero e transformadoras de gênero.

**Resultados.:**

O seminário contou com 54 participantes provenientes de 19 países. Do número total de participantes, 41 responderam ao menos uma das três partes da pesquisa, sendo que 26 responderam a parte sobre gênero. Dentre estes, 17 (65,4%) haviam conduzido coleta de dados desagregados por sexo e 14 (53,8%) haviam realizado análise de gênero em pesquisas recentes sobre o ambiente alimentar. Um pequeno número informou ter incluído os achados relativos ao gênero em recomendações e soluções. As dificuldades encontradas pelos participantes foram limitação metodológica e de dados (como a falta de evidências preexistentes e o uso de dados secundários), lacunas de conhecimento e capacidade, sensibilidade do assunto e vieses. Os tópicos de pesquisa identificados para melhorar a equidade de gênero foram normas para o preparo dos alimentos e responsabilidades domésticas, participação diferencial de homens e mulheres na produção, distribuição e comércio a varejo de alimentos e políticas escolares e de trabalho.

**Conclusões.:**

O estudo indicou que a iniquidade de gênero não está sendo devidamente contemplada em pesquisas sobre ambiente alimentar na região da ALC. A estrutura analítica aqui exposta pode servir como um importante ponto de partida e recurso para incentivar futuras pesquisas transformativas de gênero. Outros esforços são necessários para vencer os desafios mencionados pelos pesquisadores que participaram do estudo, como lacunas de capacidade, limitação de dados e recursos e barreiras à publicação dos trabalhos.

The United Nations 2030 Agenda for Sustainable Development calls for a gender-responsive implementation of the Sustainable Development Goals (SDGs) (1). Gender inequality is a significant barrier to human development, and, as such, any and all sector-specific SDG targets should “recognize women’s equality and empowerment as both an objective and a solution” (2). Despite high-level acknowledgment of the significance of gender in the epidemic of noncommunicable diseases (NCDs), including with the 2011 United Nations Political Declaration, gender continues to be neglected in the global response (2, 3).

NCDs are chronic, prolonged illnesses that result from multiple genetic, physiological, environmental, and behavioral factors rather than an infectious process. Development agencies and research institutions have been called on to fund studies that incorporate sex and gender from the initial design through to the analysis and interpretation of findings, in order to refine our understanding of gendered patterns of NCD risk factors, health-seeking behavior, and impacts (4). However, lack of rigorously analyzed sex-disaggregated data (which is the first step and prerequisite for gender analysis (5)) is an ongoing gap in NCD research in low- and middle-income countries (LMICs) (6, 7). Lack of attention to the gendered dimensions of NCDs hinders our ability to respond to the differentiated health and social impacts among women and men, and apply solutions that contribute to improved health and gender equality.

The world’s increasing capacity to produce, process, and trade food has been accompanied by important changes in dietary patterns. In recent decades, diets have shifted towards increased reliance on ultra-processed, ready-to-consume foods that are nutritionally poor, and high in fat, salt, and sugar (8, 9). In all regions of the world, unhealthy diets are one of the four major risk factors driving the rise in NCDs (10). Recent studies of Latin America and the Caribbean (LAC) have found that more than 50% of the population is overweight (11), that about 8% of the population has diabetes (12, 13), and that cardiovascular disease is the leading cause of death (14). In some countries, such as Belize and Nicaragua, overweight/obesity among women is as high as 70% (15, 16).

Research on healthy food environments is an emerging field within the context of NCD prevention. This field investigates how personal and external influences increasingly affect what people consume and what their nutritional status is. Food environments are characterized by the types of food that are available, affordable, convenient, and desirable in a given place (17). Interventions for healthier food environments lie largely outside the health sector. The interventions include fiscal measures, policies, and regulations that target the seven key domains of the food system: food composition, labeling, marketing, provision, retail, prices, and trade (18). It is important to note that interventions aimed at promoting healthier food environments in LMICs exist within gendered sociocultural environments, where women are disproportionately responsible for meal preparation and the family’s health (5, 19). Consequently, these interventions have the potential to ameliorate or reinforce gender inequities (20). Evidence is needed to inform “gender-specific” and “gender-transformative” interventions for NCD prevention. Gender-specific interventions are ones that take into account gender norms, roles, and relations and respond to the specific needs of women and men. Gender-transformative interventions go further, tackling the root causes of gender-based health inequities (21).

Countries in Latin America and the Caribbean have been at the forefront of global and regional action to tackle food-related NCDs, with research playing a significant role in informing policies and interventions (22, 23). Recent, evidence-informed interventions in the LAC region include improved nutrient labeling on packaged food, laws restricting TV food advertisements to children, and impact assessments of sugar-sweetened beverage taxes (24, 25). Pioneering research from the LAC region is already informing approaches elsewhere, and thus the presence or absence of gendered evidence is likely to have a ripple effect on the field.

This paper describes the results of a study conducted by Canada’s International Development Research Centre (IDRC) and the Brazilian Institute of Consumer Protection (Instituto Brasileiro de Defesa do Consumidor, IDEC) concerning approaches for strengthening the way gender is addressed in research on healthy food environments for NCD prevention. The objectives of the study were to examine current practice and barriers faced by LAC researchers in addressing gender in research on healthy food environments, and to identify future topics for gender-sensitive and gender-transformative research.

## MATERIALS AND METHODS

IDRC and IDEC carried out a two-part study involving: 1) a descriptive survey to investigate to what extent participating researchers from LAC are integrating gender considerations in research for healthier food environments and 2) a participatory workshop to coproduce ideas for future gender-sensitive and gender-transformative research (26, 27). IDRC funds LMIC-led research in developing countries to promote growth, reduce poverty, and drive large-scale positive change. Since 2010, IDRC has funded over 30 research projects worth more than Can$ 20 million on healthier food environments. As one of the largest funders of research on NCD prevention in LMICs, IDRC is interested in understanding how it can support more gender-responsive and gender-transformative research as part of its organizational commitments.

IDRC partnered with IDEC to collaboratively carry out this study. IDEC is a nonprofit, civil society advocacy organization that presses industry and government to adopt policies for healthier food environments. IDEC conducts independent research to inform its policy proposals and has received past funding from IDRC.

In June 2017, IDRC and IDEC hosted a three-day workshop in Brazil with the aim of enhancing networks and collaborations among food environment researchers from across Latin America and the Caribbean, and to identify research and capacity gaps in the LAC region.

Workshop participants were recruited by invitation based on subject expertise and geographic representation across LAC countries. Participants were primarily researchers previously supported by IDRC, though many also receive funding from multiple sources (e.g., country funders, bilateral donors, nongovernmental organizations (NGOs), and United Nations agencies). Representatives from a number of other key groups (e.g., Pan American Health Organization, Food and Agriculture Organization, International Network for Food and Obesity/Non-communicable Diseases Research, and Monitoring and Action Support (INFORMAS)) also attended.

Prior to the workshop, a three-part electronic survey (e-survey) consisting of open- and closed-ended questions was sent to participants, seeking information on: 1) past and present food environment research undertaken, 2) strategies for research impact, and 3) experiences integrating gender in food environment research. Convenience sampling was used and included all participants attending the workshop. Responses were not limited to research projects funded by IDRC, and included experiences across multiple projects and funders. Responses were accepted in Spanish, Portuguese, and English and were posted on an e-platform to enable participants to view the collection of responses as part of a collective and shared learning forum. We calculated descriptive statistics (e.g., frequency, means) for closed-ended questions and used inductive coding to categorize barriers listed in the open-ended questions (28). Two investigators from IDRC and IDEC jointly analyzed the preliminary findings.

As a second step, we used a participatory workshop to explore potential topics for gender-transformative research within the domain of healthy food environments. Participatory workshops are a research methodology that is well suited for producing data in emerging domains, such as gender and healthy food environments, where relatively little work has been undertaken (26). An international NGO, Gender at Work, delivered the session on gender equity using an evocative, participatory methodology to engage participants in a difficult topic. Using an analytical and conceptual tool developed by Gender at Work (available at https://genderatwork.org/analytical-framework), participants worked in groups to propose future research topics for gender-sensitive and gender-transformative studies on healthy food environments. (More information on raising awareness of and developing skills in gender analysis and gender-responsive planning, including using a gender responsiveness assessment tool, is available in a World Health Organization (WHO) manual (21)). During this session, the results of the e-survey were presented to participants and additional insights were then integrated into the findings. The workshop was a participatory event, intended to advance the field rather than assess work funded by IDRC.

### Ethics

Prior to the workshop, IDEC sent e-mails inviting workshop attendees to participate in an e-survey/e-platform for the purposes of learning and exchange. Participation was voluntary. Participants were aware that the information provided was part of a public exchange where information would be viewable, identifiable (participants posted their names and contact information), and discussed at the workshop.

Participants modified and added additional information throughout the workshop. Given that the data was provided for the purpose of sharing, no additional consent or ethical approval was sought. This is in line with current practice for online research (29, 30). Nonetheless, usernames and identifying details have been omitted for this paper.

## RESULTS

Fifty-four participants (65.8% female), from 19 countries, attended the workshop. Of those 54, 41 of them (75.9%) responded to at least one section of the three-part survey. Twenty-nine of the 41 (70.7%) participants were primary investigators on food environment research projects, of which 36.6% were solely funded by IDRC, with the rest receiving funding from multiple sources.

### Collection and use of sex- and gender-related findings in food environment research

The response rate was lower for the survey section on gender, with 26 of 41 (63.4%) of participants (77.0% female) providing responses. Of the 26, 17 of them (65.4%) indicated that they collected sex-disaggregated data, and 14 of the 26 (53.8%) indicated that they conducted sex and gender analysis within the context of a current or recent research project on food environments. The participants provided examples of novel findings related to differential risks and impacts of interventions, such as increased probability of unhealthy food consumption among teenage girls, different sources of sodium consumption (fast food, packaged foods) among men and women, and greater use of menu labeling by women. However, only 2 of the 26 respondents provided a concrete example of how the findings from sex and gender analysis informed their recommendations and solutions.

### Challenges and barriers

Respondents identified barriers and challenges to examining gender equity in food environment research (Table 1). The survey responses were echoed and elaborated on during the workshop. Nearly a quarter of participants (6 of 26) cited limited data and lack of evidence in the literature as a barrier. For example, the lack of published evidence on how the food industry targets boys versus girls or on the differential impacts of the school food environment on girls and boys led to assumptions of gender neutrality and failure to integrate gendered research questions in some projects. For example, one male participant stated:

**TABLE 1:** Challenges and barriers experienced by Latin American and Caribbean (LAC) researchers in examining gender equity in research for healthier food environments, based on a survey of LAC researchers at a 2016 workshop

Types of challenge	Challenges cited by LAC researchers
Data and methodological limitations	Gender not identified as a variable in the existing literature Gender not captured in secondary data Collecting household-level primary data not always feasible Large household surveys may not provide much insight into household-level gender dimensions Challenges in engaging male caregivers, and lower participation of men in research studies Lack of training and resources (time, money) for community-based participation and research Absence of data on sexual orientation
Lack of knowledge and capacity	Lack of knowledge on how to approach gender issues within the context of research for healthier food environments Difficulty in determining differences due to sex versus gender
Subject sensitivity	Cultural barriers that reinforce gender roles in interventions Avoiding perceptions of discrimination when identifying gendered solutions Not always feasible to invest sufficient time to build trust and relationships required to investigate gender relations in depth
Assumptions and biases	Perception among workshop participants that men and women do not significantly experience different motivations, preferences, and barriers

***Source:*** Prepared by the authors, based on the study results.

“We started our research from an understanding that unhealthy school food environments more or less equally affect boys and girls because we do not have more fine-grained data to indicate if and how gender affects one’s experience of, or navigation of, the school food environment. Gender has not been a primary focus for the study, because in the existing literature it is not explicit that gender is a key variable determining access to healthy food in schools or in the way in which children negotiate school food environments.”

Another important data limitation noted in the survey was the lack of gender variables in secondary datasets, which limited the ability of participants to investigate gendered dimensions and impacts. In the workshop discussion, participants echoed the issue of dataset limitations, noting particular challenges for economic modeling. One participant expressed frustration at having had a paper rejected because there was no basis in the literature for including gender as a factor within the economic model presented. Participants highlighted constraints around collecting more gender-sensitive data, noting that it is not always feasible to carry out new household surveys and that large household surveys do not necessarily provide in-depth insight into gender dimensions. During the discussion, other participants suggested the possibility of integrating household survey questions related to gender perceptions and division of household tasks. Other challenges for research design included the lack of resources for community-based research, the time required to build trusting relationships required for in-depth exploration of gender factors, and issue sensitivity.

Lack of knowledge, experience, and capacity were identified in both the survey and workshop discussion. Participants conveyed the need for greater sensitization among researchers to challenge assumptions of gender neutrality, and to consider gender from the time of project conception. One study participant asked if assumptions and biases among researchers are contributing to gender-blind studies: “Maybe the idea that all men and women have the same motivations, perception of barriers, and, sometimes, preferences makes researchers perform interventions without segmenting men and women.”

Among the other challenges noted were the lower recruitment of men into research studies, less access to male caregivers, absence of data on sexual and gender orientation, and the perception that gender is not relevant in some studies, such as in analyses of food composition data.

### Opportunities for gender-responsive and gender-transformative research

**FIGURE 1:**
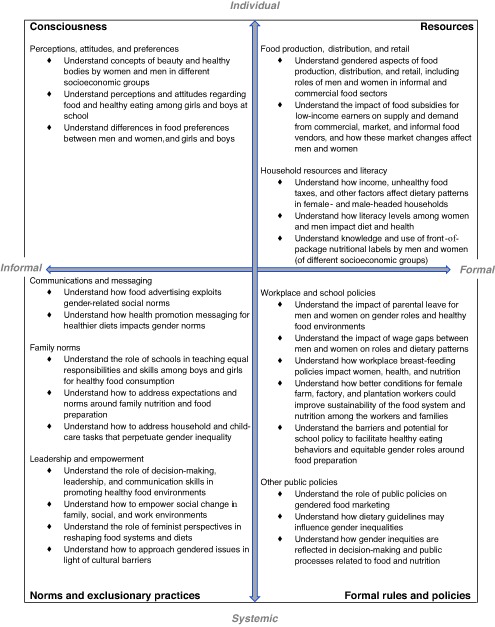
List of topics for potential gender-responsive and gender-transformative research topics on healthy food environments, as developed by Latin American and Caribbean researchers attending a 2016 workshop

***Source:*** Prepared by the authors, based on the study results.

In order to tackle gender inequities, there is a need to identify research questions and approaches that can contribute to gender-responsive and gender-transformative research. Challenges around elucidating relevant questions were voiced by many of the study participants and is an important barrier faced by the field. To this end, we employed a tool developed by Gender at Work in order to assist participants in making visible the dimensions of gender inequality within food environment research. The tool has been widely used by community and international organizations, including Oxfam International and the Global Fund for Women, and it has been cited in the academic literature (31). Using the framework, participants articulated entry points for gender-responsive and gender-transformative research that addressed: 1) informal norms and exclusionary practices, 2) consciousness, 3) resources, and 4) formal rules and policies. Responses ranged from the individual to the systemic level, with illustrative examples presented in Figure 1.

Participants raised the importance of understanding how norms and expectations around food and food preparation are established and can be changed. Among the areas of potential research that were identified were understanding how to address dynamics around domestic responsibilities and women’s empowerment, as well as understanding how food advertising and public health messaging are targeted at and exploit women, men, boys, and girls. Research questions around resource and power disparities formed a second cluster of inquiry, and included topics related to differential participation of men and women across food production, distribution, and retail (in formal and informal sectors), as well as in the policy processes shaping the food system. A third thematic cluster focused on the role of school and employment policies, including the impact of parental leave, wage gaps, work conditions for farm/plantation laborers, breast-feeding policies, and school food programs and curriculum in relation to healthy diets and gender roles. Participants also noted the importance of addressing intersectionality, that is, the way in which gender intersects with income, race, education, and other variables that can create overlapping levels of disadvantage in the context of healthy diets. In discussing the challenges, constraints, and opportunities, participants suggested the need to strengthen the ways in which gender-responsive and gender-transformative methods and questions are solicited and approached in research funding calls.

## DISCUSSION

Meaningfully investigating gendered dimensions and biases is important for scientific rigor, excellence, and maximal effect, including equitable impact, according to Tannenbaum et al. (32). Extending from that implementation research, in an internal report commissioned by IDRC, Bilkis Vissandjee highlighted the importance of gender-responsive approaches in food environment interventions: “In different cultures, women, men, and people of diverse gender identities differ in their roles, responsibilities, access to and management of resources, resilience, and participation in decision making. Because these gender differences influence a community’s distribution of the resources in their food system, it is important that programs and projects are gender responsive and aware of the potential for inequality.” (That report is available upon request by emailing feh@idrc.ca).

Our paper presents shortcomings and barriers identified by food environment researchers in integrating sex- and gender-based analysis and proposes potential future research topics for gender-responsive and gender-transformative research in the field. We believe this is the only published piece to date that presents a forward-looking agenda for gender-transformative food environment research, and that it will serve as an important tool for assisting researchers in developing future research topics that contribute to improved gender equity.

In this study, the participants highlighted a lack of existing evidence on the gendered dimensions of food environments as an important barrier, and a contributor to the ongoing neglect of gender in the design and publication of new studies. For example, study participants made assumptions of gender neutrality in research on school food environments and on food marketing to children based on the absence of evidence on gender impacts reported in the literature. However, examples provided by study participants demonstrate that gendered exposures and impacts are often present when investigated. Given the reliance on secondary data in many studies, collecting gender-specific information in household surveys, such as on household tasks, needs to become commonplace. Explicit calls by NCD-relevant journals for publications investigating sex and gender, such as the recent call by the *Journal of Studies on Alcohol and Drugs*, may be helpful in catalyzing evidence production (33).

Gender must become integral to all strategies, policies, and programs related to NCD prevention and treatment. While it was common for the participating researchers in this study to conduct some level of sex- and gender-based analysis, gender-related findings were rarely integrated into their recommendations and solutions. Reviews of other NCD-related research, including on cardiovascular health and alcohol consumption, note similar challenges in moving beyond superficial analysis of sex and gender (33, 34). Without better connecting gendered findings to interventions, we will continue to miss opportunities to address gender inequity.

Indicative findings from this study suggest that food environment research from the LAC region may largely fall under the WHO Gender Responsive Assessment Scale classification of “gender-sensitive,” in that the research considers gender norms but does not take action to redress inequities (21). The research community needs to explore how to engage in gender-specific and gender-transformative research within the context of field-specific topics, such as food advertising, food labeling, and taxes on unhealthy foods. Preliminary thinking by study participants suggest there is a range of potential entry points and opportunities, including addressing gendered targeting by the food industry and in public health campaigns, norms around domestic responsibilities, gendered roles and responsibilities at different points in the food system, and work- and school-related policies and practices. Such research should also take into account intra- and intercountry contexts that impact gender dynamics and outcomes.

We call upon the research community to expand and further the initial thinking presented in our study. A systematic review of gender-integrated health programs in LMICs (35) identified overarching strategies used in gender-aware and gender-transformative health programs that may be applicable to the field of healthy food environments. These strategies included challenging gender norms, roles, and dynamics; empowering disadvantaged groups; fostering social and behavioral change; promoting equitable relationships and decision-making; and empowering women and girls through economic opportunities, education, and collective action.

Gender is often not central in research studies and tends to come as an afterthought in response to donor funding requirements. The participatory workshop methodology led by Gender at Work helped present the activity as an intellectual challenge and produced a large number and range of contributions for potential gender-specific and gender-transformative research. Generating curiosity among researchers may be an important starting point. The workshop participants, in fact, surprised themselves with the extent to which they were able to challenge assumptions and spark new ideas where they had previously thought gender was not a relevant or interesting consideration. In the workshop, the participating researchers, stakeholders, and donors found peer learning and challenge to be an important means of pushing and expanding their thinking.

### Limitations

The study was limited by several factors, including: 1) the small sample of LMIC researchers attending the workshop, who were from a single region and who were largely funded by IDRC; 2) higher study participation by women than by men; and 3) reliance on participant self-reporting and recall. A larger and more representative sample might yield more diverse responses (including from men), while analysis of their research projects and associated publications would be useful for triangulating findings. However, with relatively few funders and LMIC researchers working on healthier food environments, the participants who attended the workshop represent a significant segment of the field, and include individuals with strong track records of policy impact and leadership. While responses could be favorably biased in light of the donor’s role in the workshop, the participants raised important challenges and mentioned crucial gaps.

Further studies are needed to understand how research funding calls, donor requirements, and other mechanisms can more effectively stimulate research communities to prioritize gender equity within public health research for NCD prevention. Understanding regional and country differences, as well as experiences of lesbian, gay, bisexual, transgender, intersex, and queer (LGBTIQ) groups, would also help to further the field.

### Conclusion

The United Nations 2030 Agenda for Sustainable Development enlists the global community to consider equity at the forefront of all action. Gender equality is considered “an enabler and accelerator for all the Sustainable Development Goals (SDGs)” (1). However, addressing inequitable gender norms and outcomes does not commonly form a key objective in food environment research from the LAC region. The analytical framework presented in this study provides an important tool for assisting researchers in developing topics that address the root causes of gender inequity. Complementary actions are needed by funders, publishers, and other actors in the public health field to overcome other barriers related to the lack of knowledge, motivation, and skills among researchers; resource limitations for data collection; and the lack of opportunities to publish gender-sensitive and gender-transformative research on healthy food environments.

## Author contributions.

TL conceived the original idea, planned the study, collected and analyzed the data, interpreted the results, and wrote and reviewed the paper. DN planned the study, collected and analyzed the data, and reviewed the paper. TL and DN reviewed and approved the final version.

## Acknowledgments.

We are grateful to all our colleagues at Canada’s International Development Research Centre (IDRC), the Instituto Brasileiro de Defesa do Consumidor (IDEC), and Gender at Work for their insights and contributions to delivering the workshop and preparing this publication. This is particularly true for Rex Fyles, Carly Hayes, Ana Paula Bortoletto, Natacha Lecours, Zee Leung, Greg Hallen, and Lais Amaral Mais. We would also like to thank all the workshop participants for sharing their experiences and insights.

## Funding.

This study was carried out as part of operational activities supported by IDRC’s Food, Environment, and Health program.

## Disclaimer.

The authors hold sole responsibility for the views expressed in the manuscript, which may not necessarily reflect the opinion or policy of the *RPSP/PAJPH* and/or PAHO.
